# An observational study of the factors associated with frequency of outpatient benzodiazepine prescribing to patients receiving chronic opioid analgesic therapy in primary care at a major academic center

**DOI:** 10.1186/s12875-022-01936-z

**Published:** 2022-12-13

**Authors:** Nancy V. Koch, Richard J. Butterfield

**Affiliations:** Division of General Internal Medicine (Koch) and Division of Clinical Trials and Biostatistics (Butterfield), Mayo Clinic, 13400 E Shea Blvd, Scottsdale, AZ USA

**Keywords:** Alcohol, Anxiety, Chronic pain, Elderly, Falls, Outpatient practice, Patient safety, Prescription drug abuse, Primary care, Psychiatry

## Abstract

**Background:**

Prescribing benzodiazepines to patients taking chronic opioid analgesic therapy increases risks of adverse events. In 2016, the Centers for Disease Control and Prevention recommended avoidance of benzodiazepine prescribing concurrently with opioids, and various organizations have instituted similar guidelines. We aimed to determine the frequency and patterns of benzodiazepine prescribing at Mayo Clinic primary care (Community Internal Medicine, Family Medicine) clinics for patients taking chronic opioid analgesic therapy and the characteristics of patients receiving the prescriptions and providers administering them.

**Methods:**

This retrospective observational study included adult patients taking chronic opioid analgesic therapy for 2 full years in 2018 and 2019 at Mayo Clinic primary care practices in Arizona and Florida. We assessed electronic health records for these individual patients to determine whether they received a benzodiazepine prescription during the study period and how frequently they received a prescription. Variations in prescriptions by provider specialty, location, and sex were studied. Documented data included receipt of a benzodiazepine prescription by patients with at-risk alcohol use or alcohol use disorder, depression, anxiety, chronic obstructive pulmonary disease, falls, and psychiatric referral. Data were compared between patients who received benzodiazepines and those who did not with the Kruskal-Wallis test or χ^2^ test, and the Wilcoxon signed rank test was used to assess whether the change in number of benzodiazepine prescriptions (2018 vs. 2019) was different from zero.

**Results:**

Study participants (*N* = 457) were predominantly women (*n* = 266, 58.2%); median age was 69 years. In total, 148 patients (32.4%) received benzodiazepine prescription. These patients were more likely to be women (*P* = .046) and younger (*P* = .02). Mean percentage change was 176.9% (*P* < .001) in number of benzodiazepine prescriptions provided from 2018 to 2019. Frequency of referral to mental health providers was low, as was presence of an established mental health provider despite a greater prevalence of anxiety (*P* < .001) and depression (*P* = .001) among patients receiving benzodiazepines.

**Conclusion:**

Benzodiazepine prescription to individual patients taking chronic opioid analgesic therapy significantly increased from 2018 to 2019 despite the documented risks and harms associated with such practice. No statistically significant difference was observed in frequency of benzodiazepine prescriptions between practice location, sex of provider, or specialty.

## Background

In 2019, 16% of overdose deaths involved benzodiazepine (BZD) use [1]. The overdose death rate was higher for patients taking the combination of BZDs and opioids. Statistics show that the number of adults filling a BZD prescription increased by 67% from 1996 to 2013, from 8.1 million to 13.5 million prescriptions. In this same period, the quantity of lorazepam equivalents per 100,000 adults increased from 1.1 kg to 3.6 kg. [1].

Several medical organizations have since written guidelines advising against coprescribing BZDs to patients taking chronic opioid analgesic therapy (COAT) [2–7]. The use of BZDs with opioids for older patients with chronic pain can increase risks of overdose, respiratory depression, falls, mental health disorders, cognitive impairment, and hip fractures [8–15]. Risks of respiratory depression and death with this combination is particularly dangerous in older patients with chronic obstructive pulmonary disease [16].

BZDs are often prescribed to patients with at-risk alcohol use or alcohol use disorder who receive COAT for pain. Serious impairment of the central nervous system can occur because of the synergistic sedative effects of BZDs and alcohol. Because the metabolism of some BZDs and alcohol involves cytochrome P450, the decreased metabolic rates of these substances can have adverse or fatal consequences [17]. Coinvolvement of alcohol and BZDs in opioid overdose deaths in 2017 was 14.7% for alcohol and 21.0% for BZDs [18]. In another study, patients with chronic pain who were diagnosed with alcohol use disorder showed a trend toward longer opioid use [19]. A study by University of California San Francisco and Kaiser Permanente in Northern California showed that BZD use in primary care was associated with unhealthy alcohol use [20]. The Drug Abuse Warning Network analyzed drug-related emergency department visits from 2005 to 2011, reporting that BZDs prescribed with opioid pain relievers or taken with alcohol increased the risk of serious visit outcomes by 24–55% compared with BZD alone [21].

Little detail has been documented about BZD prescription to patients taking COAT with underlying risk factors and whether the recommended guidelines against concomitant use have resulted in decreasing such coprescribing [22–26]. The purpose of the present study was to assess the frequency of concomitant BZD prescribing in 2 recent years for patients receiving COAT at primary care outpatient sites and to evaluate for patient and provider factors that place patients at higher risk for overdose.

## Methods

The present study was performed to evaluate the percentage of patients who took COAT and received BZD prescriptions at various outpatient primary care Mayo Clinic practices in Arizona and Florida from January 1, 2018, through December 31, 2019. We defined *COAT* as episodes of opioid prescribing lasting longer than 90 days and a total supply for 120 or more days. The investigation included the number of BZD prescriptions written at these practices. We analyzed the variation in BZD prescribing by patient age and sex, sex of provider, practice location and specialty, and the presence of harmful alcohol use (defined as ≥ 14 drinks weekly for men and ≥ 7 drinks weekly for women) or alcohol use disorder, anxiety, depression, chronic obstructive pulmonary disease, fall risk, and psychiatry referral or establishment of a mental health clinician for patients prescribed BZDs.

The electronic health record (EHR) database system of Mayo Clinic was searched for all patients aged 18 and older taking COAT for 2 full years in 2018 and 2019 and who received BZD prescriptions in the study period. Patients were excluded if any of the following situations applied: not under care in both 2018 and 2019; BZD provided for procedural sedation; diagnosis of cancer or amyotrophic lateral sclerosis; enrollment in hospice care; enrollment in the Mayo Clinic Pain Rehabilitation Center, which involves withdrawal of all controlled substances; and transfer of care or death. For this study, prescription data monitoring sites were accessed; patient medication lists, pharmacy records, and practice refill documentation were evaluated. Patients with cancer or the diagnosis of end-stage disease and those enrolled in hospice care were excluded in accordance with current Centers for Disease Control and Prevention guidelines for patients receiving COAT [4].

EHRs were searched for presence of alcohol use that included at-risk use or alcohol use disorder; the diagnosis of chronic obstructive pulmonary disease; history of falls or documentation of fall risk; presence of anxiety or depression; and documentation of established mental health clinician or referral to psychiatry services. Risk factors were determined to be present if documented by the patient or the provider in the EHR. The Mayo Clinic Institutional Review Board approved an exemption for this study because it involved EHR review of Mayo Clinic patients.

Patient demographic, clinical, and documented comorbidity characteristics were compared between patients who received BZD prescriptions and those who did not. Kruskal-Wallis test or χ^2^ test was used where applicable. Paired change and paired percentage change in BZD prescriptions of 2018 and 2019 were assessed. Wilcoxon signed rank test was used to assess whether the change in number of BZD prescriptions was different than zero. All analyses were 2-sided, and statistical significance was considered as *P* less than 0.05. Analyses were performed with statistical software (SAS version 9.4; SAS Institute Inc).

## Results

Of 572 patients reviewed, 457 patients were included in the present study. COAT visits were performed by primary care providers at Mayo Clinic in Arizona and Florida. BZD prescriptions were provided to more than 30% of patients in the specialties, and no statistical difference was observed between these specialties (*P* = .80) (Table 1). The percentage of patients taking COAT who were prescribed BZDs was 29% or more across both sites (*P* = .09). BZD prescription was provided by an essentially equal percentage of female and male providers (32.7% vs. 31.9%; *P* = .86).

**Table 1:** Practice setting, demographic characteristics, comorbidities, and referral by provision of BZD

	BZD prescription provided^a^			
Characteristic	No (*n* = 309)	Yes (*n* = 148)	Total (*N* = 457)	*P* value
**Practice setting**				
Service name				.80^b^
Community Internal Medicine	150 (68.2)	70 (31.8)	220 (48.1)	
Family Medicine	159 (67.1)	78 (32.9)	237 (51.9)	
Site				.09^b^
Arizona	203 (70.5)	85 (29.5)	288 (63.0)	
Florida	106 (62.7)	63 (37.3)	169 (37.0)	
Sex of provider				.86^b^
Female	179 (67.3)	87 (32.7)	266 (58.2)	
Male	130 (68.1)	61 (31.9)	191 (41.8)	
**Demographic characteristics of patients**				
Age, median (IQR), y	70 (61–79)	66 (59–75)	69 (61–77)	.02^c^
Sex				.046^b^
Female	170 (63.9)	96 (36.1)	266 (58.2)	
Male	139 (72.8)	52 (27.2)	191 (41.8)	
Comorbidity				
Alcohol use disorder	189 (69.0)	85 (31.0)	274 (60.0)	.45^b^
Depression	120 (59.7)	81 (40.3)	201 (44.0)	< .001^b^
Anxiety	100 (43.1)	132 (56.9)	232 (50.8)	< .001^b^
Documented fall or fall risk	99 (67.8)	47 (32.2)	146 (31.9)	.95^b^
COPD	29 (67.4)	14 (32.6)	43 (9.4)	.98^b^
Psychiatry or psychology referral or mental health provider established on record				< .001^b^
No	273 (88.3)	112 (75.7)	385 (84.2)	
Yes	36 (11.7)	36 (24.3)	72 (15.8)	

Abbreviations: BZD, benzodiazepine; COPD, chronic obstructive pulmonary disease

^a^ Values are presented as number (percentage) unless specified otherwise

^b^ χ^2^ test

^c^ Kruskal-Wallis test

**Fig. 1:**
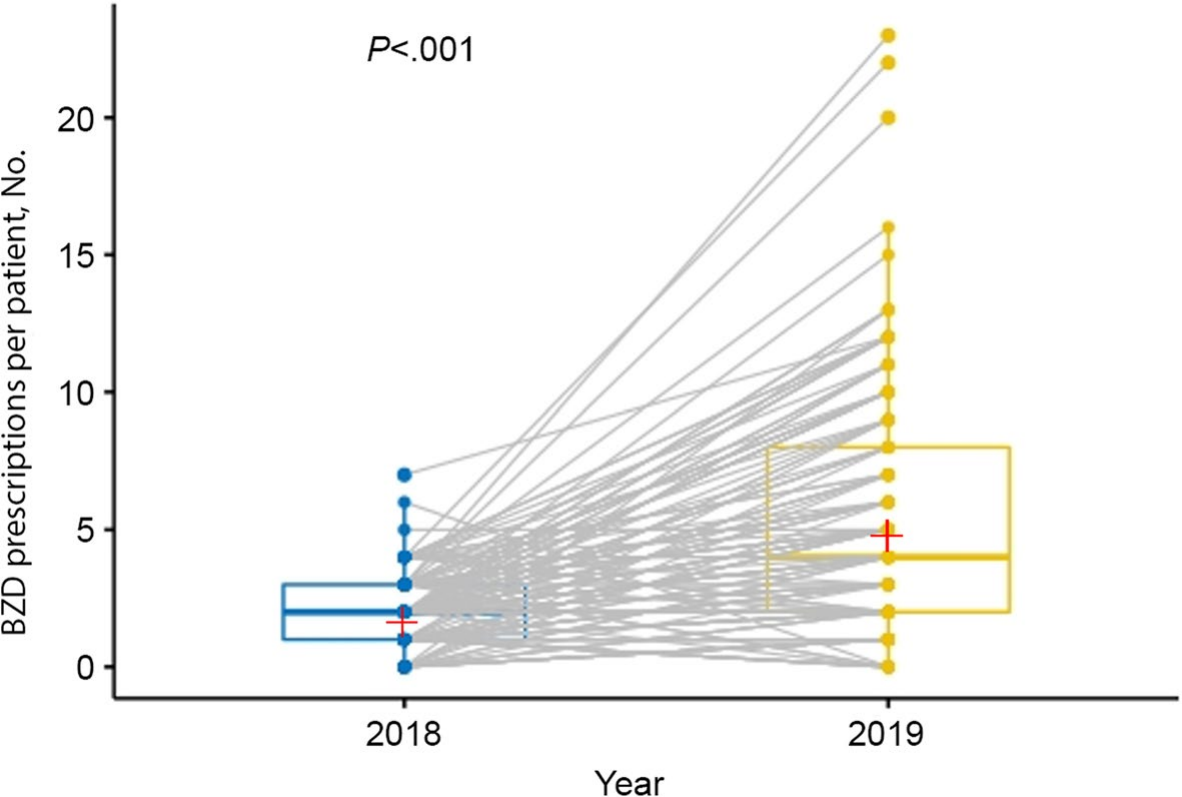
Change in Provision of Benzodiazepine (BZD) Prescriptions, 2018–2019. Data are for patients who received at least 1 BZD prescription in either year (paired *n* = 163). Values are presented as mean (SD). Wilcoxon signed rank test, *P* < .001. Plus sign indicates mean

Median age of patients taking COAT who received BZD prescription was 66 years versus 70 years for patients taking COAT alone (*P* = .02) (Table 1). A greater percentage of female patients taking COAT received BZDs (36.1% vs. 27.2%; *P* = .046). A substantially greater number of BZD prescriptions were provided in 2019 than 2018 (mean paired change, 3.5; *P* < .001), with mean paired percentage change of 176.9% (Fig. 1).

Among patients taking COAT, a BZD prescription was provided to 85 (31.0%) of 274 patients with at-risk alcohol use or alcohol use disorder, 81 (40.3%) of 201 with depression, and 132 (56.9%) of 232 with documented anxiety disorder (Table 1). Depression and anxiety disorder were significantly associated with provision of BZD (both *P* < .001). In addition, 112 (75.7%) of 148 patients who were prescribed BZD were not referred to psychiatry services and did not have an established mental health clinician on record. Psychiatry referral or a mental health clinician on record was more prevalent among patients provided BZD prescriptions than those taking COAT alone (24.3% vs. 11.7%; *P* < .001).

Of 146 patients with documented falls or fall risk, 47 (32.2%) were provided BZD prescriptions (Table 1). Yet, falls or fall risk was not found to be significantly greater for those taking COAT and prescribed BZD compared with those not prescribed BZDs (*P* = .95). BZDs were provided to 14 (32.6%) of 43 patients taking COAT who had a diagnosis of chronic obstructive pulmonary disease.

## Discussion

The risks of BZD use and dependance have been well researched and documented. However, few reports have been published on the risks of BZD prescription for patients taking COAT, despite guidelines advising against this practice. Furthermore, little information is available on the underlying characteristics of patients and providers receiving or administering these combinations. Updated guidelines have advised clinicians writing COAT prescriptions against coprescription of opioids and BZDs. Yet, the reasons for this recommendation in patients with certain risk factors for overdose have not been made clear, and the risks are not well defined. We aimed to document the underlying diagnosis and characteristics of patients taking COAT that yield higher risk of adverse events, including overdose.

This study revealed that among patients receiving COAT through primary care providers, no statistically significant difference was noted in percentage of BZD prescriptions provided by location or by sex of provider. We conclude that Mayo Clinic practices in Arizona and Florida prescribe BZDs equally to patients taking COAT. The information suggests that BZD prescribing is not affected by practice location, climate, or patient residence. Improved and more formal centralized education is needed about the risks of coprescribing, with all Mayo Clinic providers who write COAT prescriptions across the US receiving the education.

In this study, receipt of a BZD prescription by patients was associated with younger mean age and female predominance. In addition, the median age at BZD receipt was 66 years, which is 1 year after the age of Medicare eligibility and at an age when documented risks of BZD use even independently are high. This concerning clinical situation suggests that more vigilance is needed to ensure that elderly patients taking COAT are screened for BZD use regularly and, if noted, efforts made to taper from use to prevent adverse events and overdose.

Relatively younger patients taking COAT were more likely to receive BZDs compared with older patients. This information implies that younger patients with chronic pain have increased anxiety that warrants further evaluation. Significantly more women taking COAT were prescribed BZDs, suggesting that women may have or relate more anxiety with chronic pain, that providers coprescribe anxiolytics to more female than male patients, and that more female patients taking COAT have comorbid anxiety disorder.

Of note, BZDs were administered to about one-third of patients taking COAT with at-risk alcohol use or alcohol use disorder compared with patients who did not have this diagnosis. Although it was not statistically significant, this finding is important because (1) patients with long-term alcohol use should not be provided BZDs and (2) BZDs taken with COAT can cause serious cognitive impairment and respiratory depression. We believe that more patients have alcohol use disorder than what this study showed. The number of patients is underestimated because many patients do not report alcohol use or providers do not list this diagnosis in the EHR, or both. More vigilant practice is needed in screening for and documenting at-risk alcohol use or alcohol use disorder in patients taking COAT and BZDs.

Substantially more patients taking COAT with documented depression and anxiety received BZDs than those without these diagnoses. Likely, the total number of patients with these mood disorders was underestimated in the present study because many mood disorders are underreported by patients and providers. Greater transparency is needed in the recognition and treatment of concomitant mood disorders of patients taking COAT, and earlier treatment of depression and anxiety with safer and nonaddicting medications such as selective serotonin reuptake inhibitors (SSRIs) or serotonin-norepinephrine reuptake inhibitors (SNRIs) and psychotherapy may improve both mood and pain control. Most importantly, although not assessed in this study, the use of SSRIs or SNRIs may obviate the need to take substances for anxiety such as BZDs, which are shorter acting, are more addicting, and increase the risk of overdose. This should be further evaluated.

Psychiatrists and providers who specialize in addiction medicine can have a crucial role in identifying patients taking COAT who are at high risk for use of additional controlled substances such as BZDs. Several guidelines advise identification and prompt referral of these patients to behavioral health specialties to prevent overdose risk and provide treatment of mental health and substance use disorders [3,4,6].

Significantly more study patients receiving COAT and BZDs either had an established mental health provider on record or were referred to a psychiatrist than those not receiving BZDs. Yet only 24.3% of patients receiving COAT and BZD prescriptions (and 15.8% overall) were referred to or had an established mental health provider. Hence, most patients with chronic pain and mood disorders are left without good resources, treatment, and counseling. This referral barrier is likely due to the small number of available community behavioral health providers or to the fear of discussing mood or assessing for mood disorders, or both, at COAT visits. Time constraints during the COAT visits also might have a role.

Underreporting of falls continues to be an important issue in EHR documentation for patients and providers. Patients often are embarrassed to report falls and may deny any fall history when asked. Likewise, many providers neglect to ask all patients about falls because of appointment time constraints and may not report it in the EHR even when endorsed by patients.

BZD prescriptions were provided to one-third of patients with the diagnosis of chronic obstructive pulmonary disease, a diagnosis that most patients taking COAT did not have in the EHR. The number of patients with this diagnosis is likely underestimated because emphysema is diagnosed clinically with chest radiographs or pulmonary function tests, and bronchitis is rarely documented as a chronic condition. Additionally, screening is not routinely performed for asymptomatic patients at risk, including smokers. Patients with chronic obstructive pulmonary disease are at risk for adverse events, including respiratory failure, when taking opioids and BZDs concurrently [27]. Studies have shown that patients who have chronic obstructive pulmonary disease are often prescribed BZDs for breathlessness or for anxiety, especially late in the illness, and are more likely to have somnolence or chronic obstructive pulmonary disease exacerbations [28].

This study had several limitations. First, the change in frequency of BZD prescribing from 2018 to 2019 documented the number of times a prescription was written and not the actual amount or dose provided. More frequent dosing as part of a weaning protocol could have accounted for the increased number of prescriptions written; however, weaning intent was not reflected in the prescription drug monitoring program or office refill notes. Furthermore, patients could have received BZDs or BZD prescriptions from family members or providers outside our institution, which was difficult to track, so amounts and dose equivalents of prescriptions were likely greater than noted. Second, information on underlying medical disorders or risk factors could be pulled from only the EHR, which tends to underreport disease states, concomitant substance use, and falls. Third, the study included providers and patients at a large academic center with locations in multiple states. The results might not apply to providers who are in solo practice or who are not part of an academic center or to patients who do not receive COAT from academic centers. Fourth, no reason exists to believe the underestimation differs between those provided BZDs and those who were not.

Regular surveillance of patient risk factors with improved and enhanced risk assessment tools and advanced EHR methods would enhance safety and reduce harm among patients taking COAT. Safer and alternative treatments should be accessible to all COAT-administrating providers and to patients with comorbid pain and anxiety. Medications such as SSRIs and SNRIs and an expansion of interdisciplinary programs that treat mood disorders and pain could enhance healing and decrease anxiety for patients with chronic pain. The study results indicate that further investigations are needed on coprescribing of BZDs and COAT comparing our institution with other large US academic centers. In addition, study is necessary on coprescribing in alcohol use, underreporting and EHR documentation of falls and fall risk, and chronic pain with mood disorders (e.g., more frequent screening for comorbid anxiety of women taking COAT).

## Conclusion

The most important finding in this study of Mayo Clinic clinicians prescribing COAT is that BZD prescribing dramatically increased when it would be expected to decrease from 2018 to 2019—despite guidelines advising the contrary. This outcome reports that (1) guidelines alone do not change prescribing patterns and (2) more provider education is needed about the risks of coprescribing BZDs and COAT, especially to patients with underlying risk factors.

## Data Availability

The dataset supporting the conclusions of this article is included within the article (and its additional files). Raw data are available upon reasonable request from coauthor Richard J. Butterfield III, Division of Clinical Trials and Biostatistics, Mayo Clinic, 13400 E Shea Blvd, Scottsdale, AZ 85259.

## Abbreviations

BZD: Benzodiazepine
COAT: Chronic opioid analgesic therapy
EHR: Electronic health record
SNRI: Serotonin-norepinephrine reuptake inhibitor
SSRI: Selective serotonin reuptake inhibitor
